# Supplementary material for: Structural insights into the clustering and activation of Tie2 receptor mediated by Tie2 agonistic antibody
Source: Nat Commun. 2021 Nov 1;12:6287. doi: 10.1038/s41467-021-26620-1 (PMC8560823; doi:10.1038/s41467-021-26620-1)
Supplement: Supplementary file 1 — Supplementary Information [file 41467_2021_26620_MOESM1_ESM.pdf]

Supplementary Information

Structural insights into the clustering and activation of Tie2 receptor mediated by Tie2 agonistic antibody

Gyunghee Jo, Jeomil Bae, Ho Jeong Hong, Ah-reum Han, Do-Kyun Kim, Seon Pyo Hong, Jung A Kim, Sangkyu Lee, Gou Young Koh, Ho Min Kim

Supplementary Figs.1-6, Table1 and Their Legends

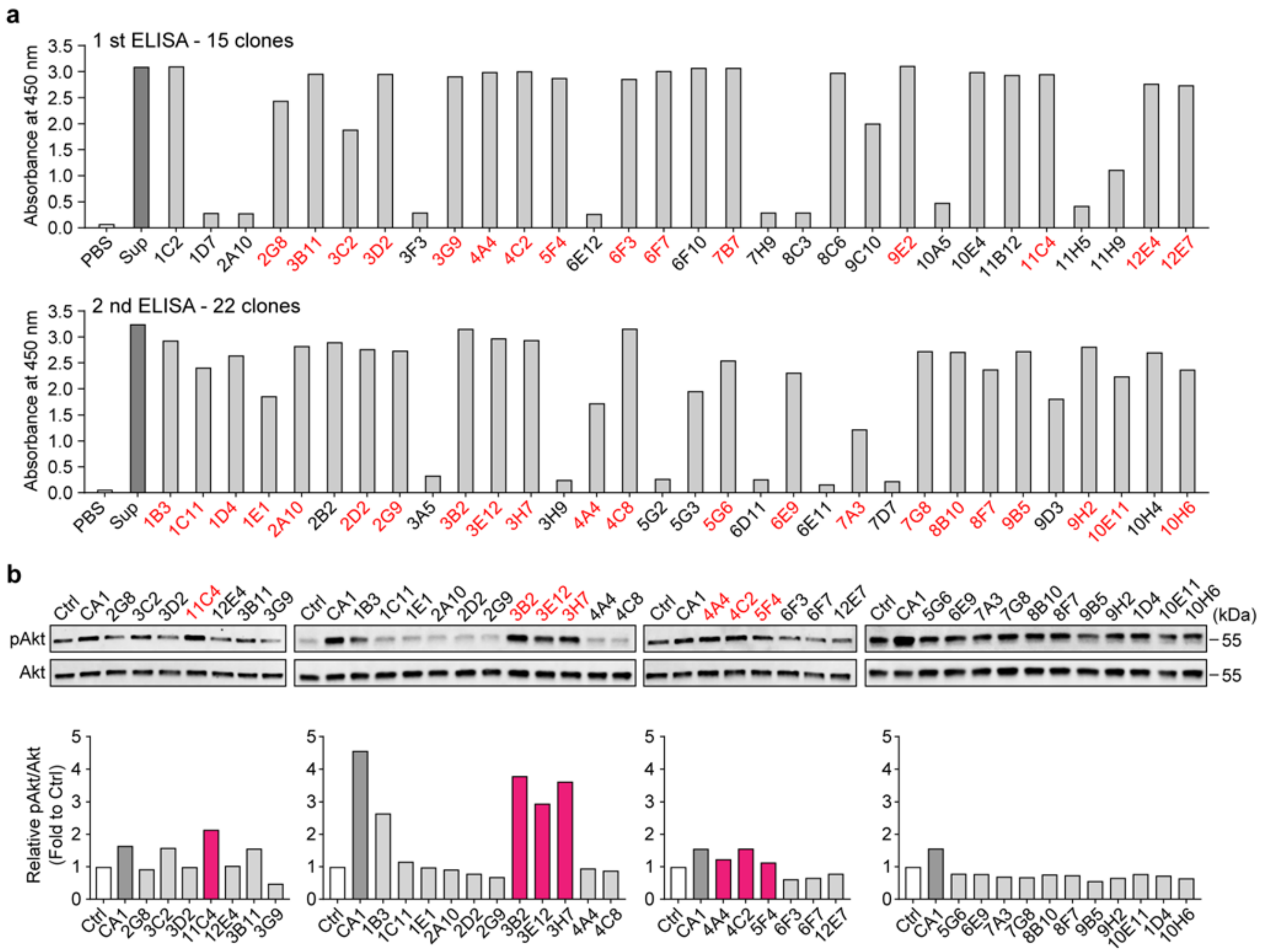

Supplementary Fig.1 | Screening of Tie2-binding and -activating antibodies

a, Clonal selection of Tie2-binding antibodies by ELISA against hTie2 Ig3-Fn3. For ELISA, 96-well plates were coated with hTie2 Ig3-Fn3 (100 ng/well), washed with PBS, and then blocked with 3% skim milk in PBS, after which 100 μl of hybridoma cell culture supernatant was added to each well and incubated for 2 hr, then washed with PBS. Bound antibodies were detected with HRP-conjugated anti-mouse IgG (1:10,000) and subsequent incubation with TMB (3,3′,5,5′-tetramethylbenzidine) solution. Total hybridoma supernatant (Sup) was used as a positive control. Selected candidate antibodies are colored in red.

b, Immunoblot detection of Tie2 downstream signaling (AKT and pAKT) in HUVECs upon treatment with each of 35 purified candidate antibodies for 30 min. Selected candidate antibodies are colored in red. No treatment was used as a negative control (Ctrl).

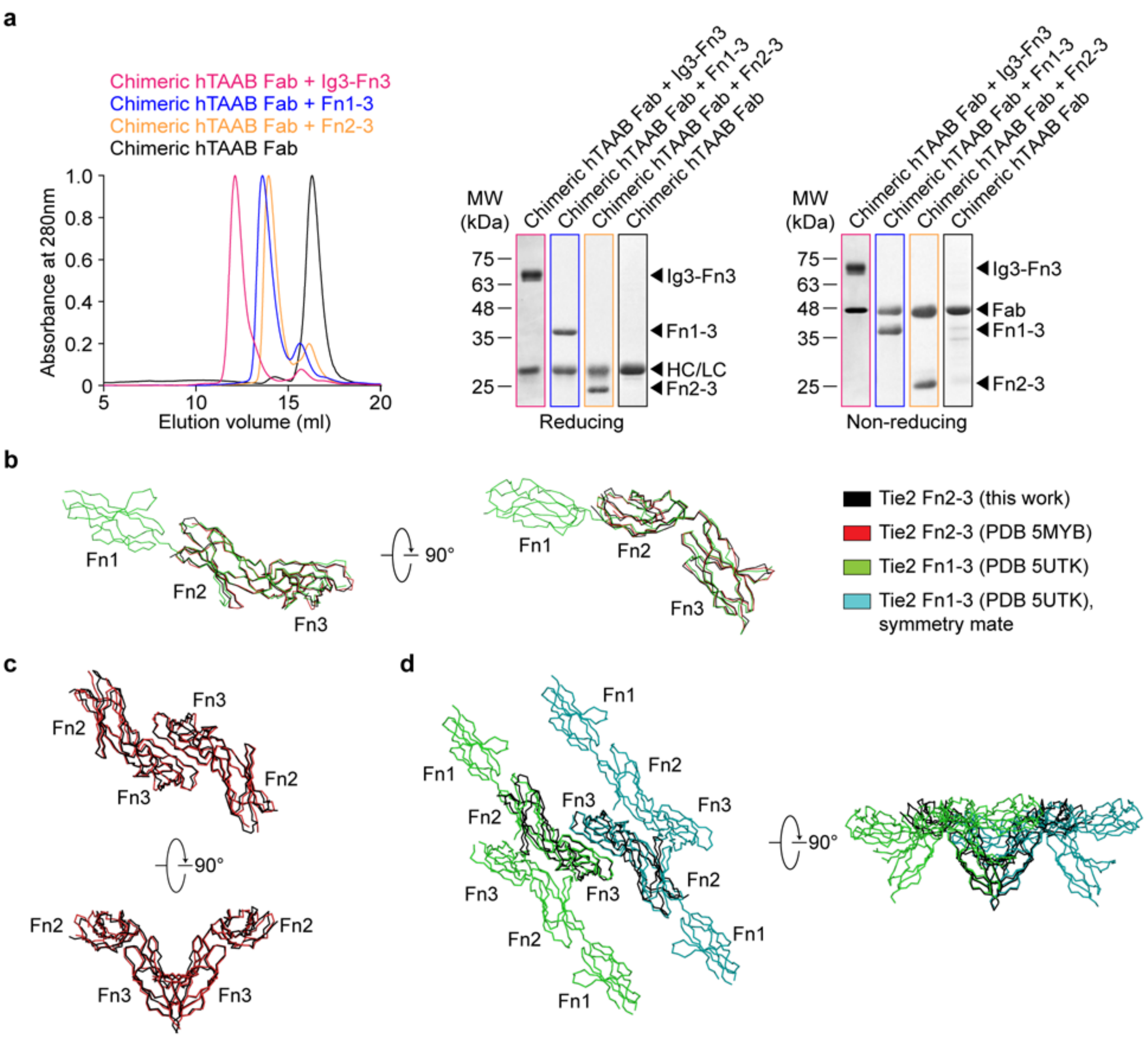

Supplementary Fig.2 | Structural comparison of Tie2 Fn2-3

a, Size-exclusion chromatography of chimeric hTAAB Fab alone (black line) and in complex with Tie2 ECD variants (Ig3-Fn3, magenta line; Fn1-3, blue line; and Fn2-3, yellow line) (left). Elution fractions were analyzed by SDS-PAGE and Coomassie blue staining under reducing and non-reducing conditions (right). Similar results were observed in three independent experiments.

b, Comparison of the chimeric hTAAB Fab-bound Tie2 Fn2-3 monomer (black) with a previously reported monomeric structure of Tie2 Fn2-3 (red, PDB: 5MYB chain A) and Fn1-3 (green, PDB: 5UTK chain A). Structures are shown as ribbon diagrams.

c, Comparison of the chimeric hTAAB Fab-bound Tie2 Fn2-3 dimer (black) with a previously reported structure of the Tie2 Fn2-3 dimer (red, PDB: 5MYB).

d, Comparison of the chimeric hTAAB Fab-bound Tie2 Fn2-3 dimer (black) with a previously reported structure of the Tie2 Fn1-3 dimer (PDB: 5UTK). The Tie2 Fn1-3 dimers in two different asymmetric units in the crystal lattice are colored green and cyan, respectively.

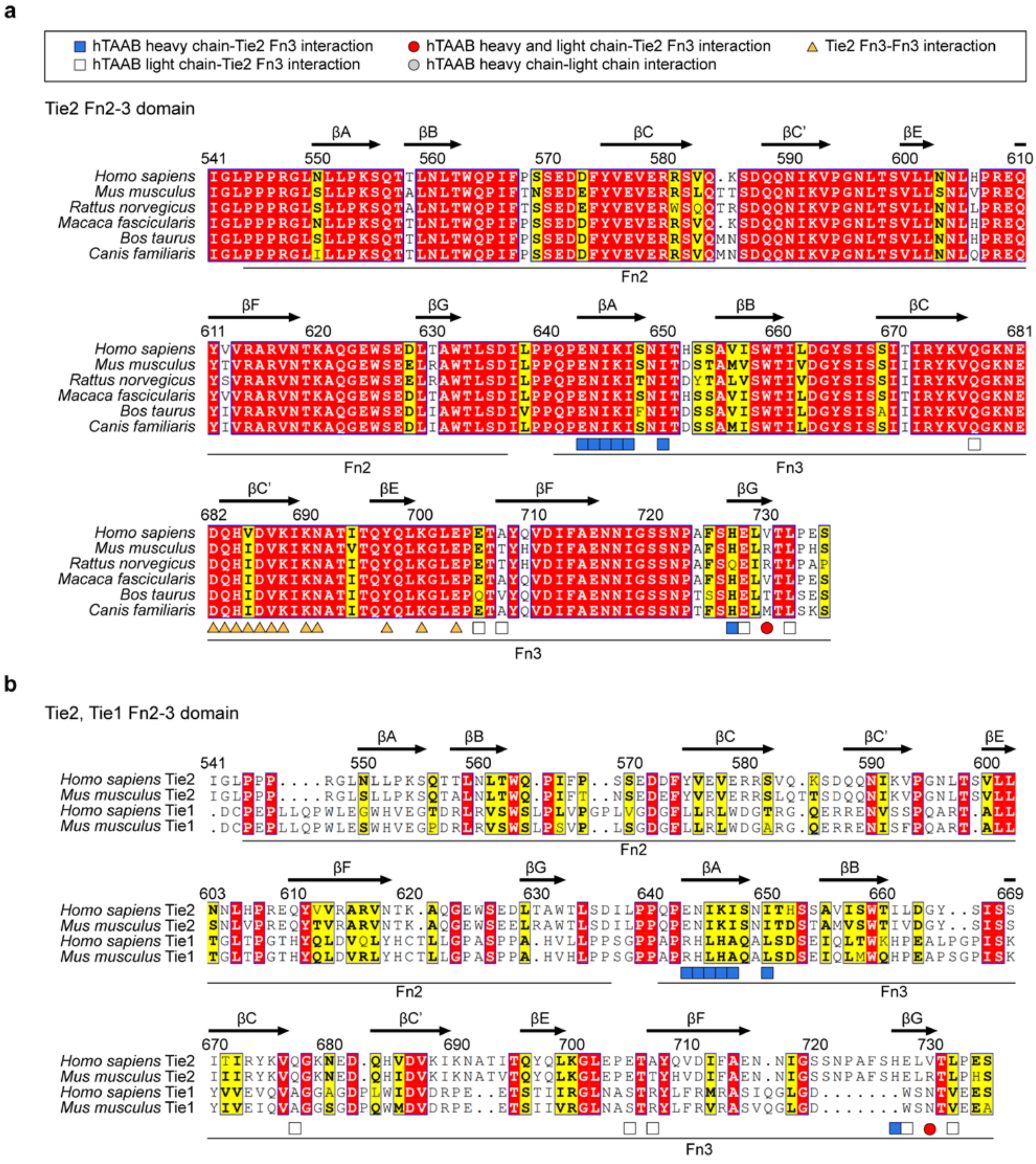
a
hTAAB heavy chain-Tie2 Fn3 interaction
hTAAB heavy and light chain-Tie2 Fn3 interaction
Tie2 Fn3-Fn3 interaction
hTAAB light chain-Tie2 Fn3 interaction
hTAAB heavy chain-light chain interaction
Tie2 Fn2-3 domain
Homo sapiens
Mus musculus
Rattus norvegicus
Macaca fascicularis
Bos taurus
Canis familiaris
Fn2
Fn3
b
Tie2, Tie1 Fn2-3 domain
Homo sapiens Tie2
Mus musculus Tie2
Homo sapiens Tie1
Mus musculus Tie1
Fn2
Fn3

(Continued)

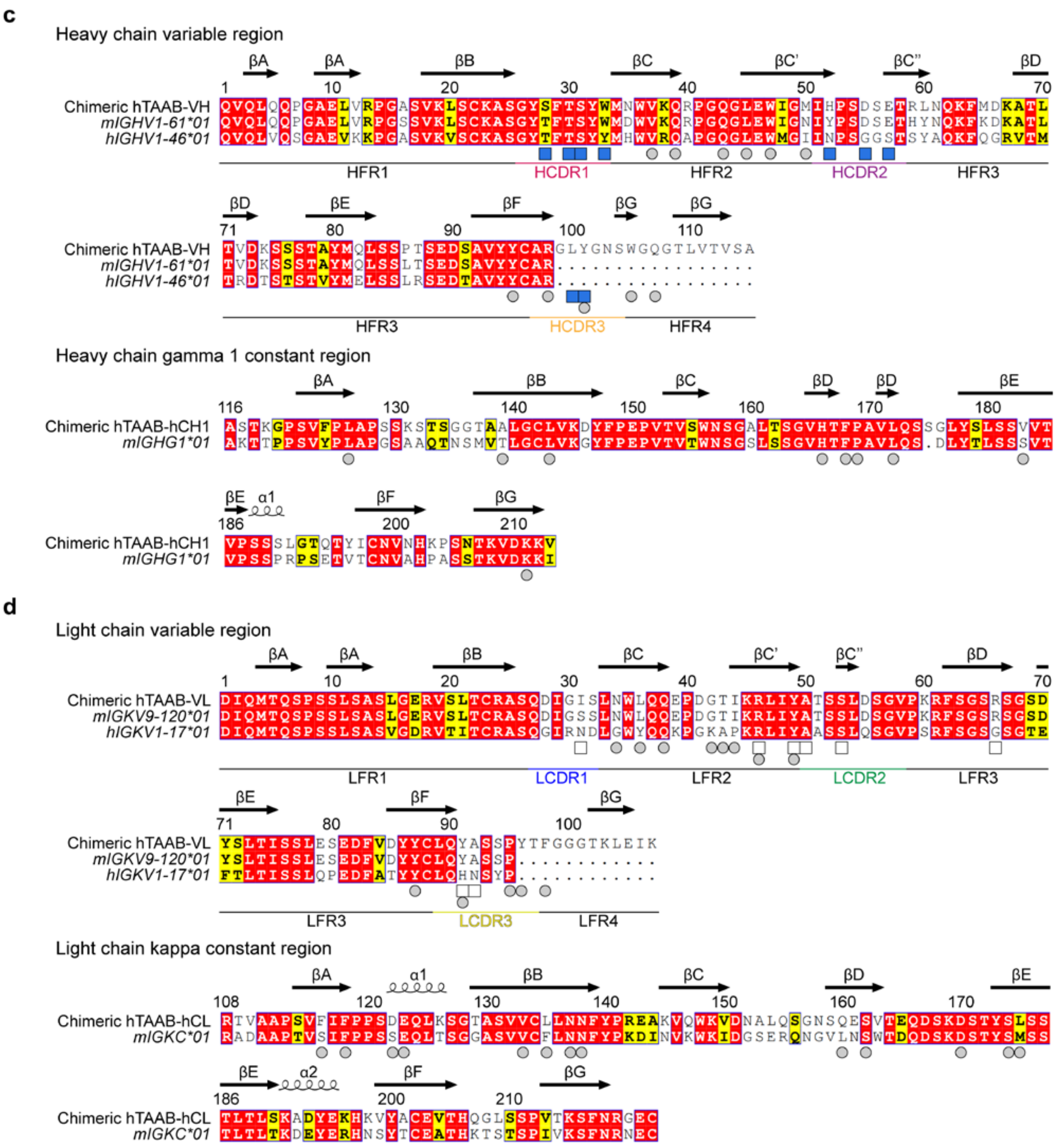

Supplementary Fig.3 | Sequence alignments of Tie2, Tie1 Fn2-Fn3 domain and chimeric hTAAB Fab

a, Sequence alignment of human (H. sapiens, UniProt: Q02763), mouse (M. musculus, UniProt: Q02858), rat (R. norvegicus, UniProt: D3ZCD0), monkey (M. fascicularis, UniProt: A0A2K5VRI3), bovine (B. taurus, UniProt: Q06807) and dog (C. familiaris, UniProt: F1P8U6) Tie2 Fn2-3.

b, Sequence alignment of human (H. sapiens, UniProt: Q02763) and mouse (M. musculus, UniProt:

Q02858) Tie2 Fn2-3, and human (H. sapiens, UniProt: P35590) and mouse (M. musculus, UniProt: Q06806) Tie1 Fn2-3.

c, Sequence alignment of heavy chain variable region of chimeric hTAAB and closest mouse and human germline genes (top), and heavy chain gamma 1 constant region of chimeric hTAAB (hIGHV1*01) and closest mouse germline gene (bottom).

d, Sequence alignment of light chain variable region of chimeric hTAAB and closest mouse and human germline genes (top), and light chain kappa constant region of chimeric hTAAB (hIGKC1*01) and closest mouse germline gene (bottom).

(a–d) Blue squares indicate interacting residues in the chimeric hTAAB Fab heavy chain and hTie2 Fn3 interface. White squares indicate interacting residues between chimeric hTAAB Fab light chain and hTie2 Fn3. The red circle indicates residue V730 of hTie2, which interacts with both heavy and light chains. Gray circles depict residues involved in interactions between the heavy chain and light chain of chimeric hTAAB Fab. The residues involved in dimeric interactions between Fn3 and Fn3 domains of Tie2 are presented as orange triangles. Red boxes indicate perfect sequence conservation, whereas yellow boxes show residues with >70% similarity based on physicochemical properties. Secondary structure elements are noted above alignment with arrows (β stands) and helix (α-helix). The sequence alignment was created using T-Coffee (http://tcoffee.crg.cat) and ESPript servers (http://espript.ibcp.fr).

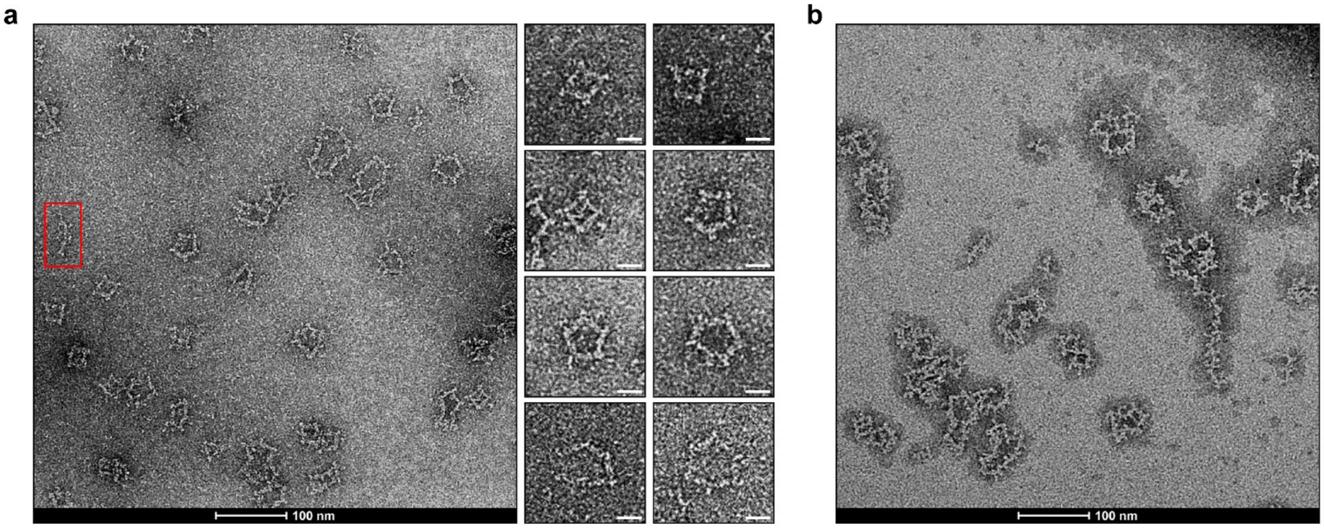

Supplementary Fig.4 | Negative-stain EM analysis of the Tie2 dimeric mutant/hTAAB IgG1 complex

a, Representative micrograph of negative-stain EM analysis of purified hTie2 Ig3-Fn3 N691C/D682C in complex with hTAAB IgG1 (left; scale bars, 100 nm). Representative particle images of tetragonal, pentagonal and hexagonal closed-ring structures. Similar results were observed in *n* = 10 micrographs from three independent experiments. Linear shape of cluster is indicated by red box (right; scale bars, 20 nm).

b, Representative micrograph of negative-stain EM analysis of aggregation peak from size-exclusion chromatography of hTie2 Ig3-Fn3 N691C/D682C and hTAAB IgG1 complex, Scale bars, 100 nm. Similar results were observed in *n* = 10 micrographs from three independent experiments.

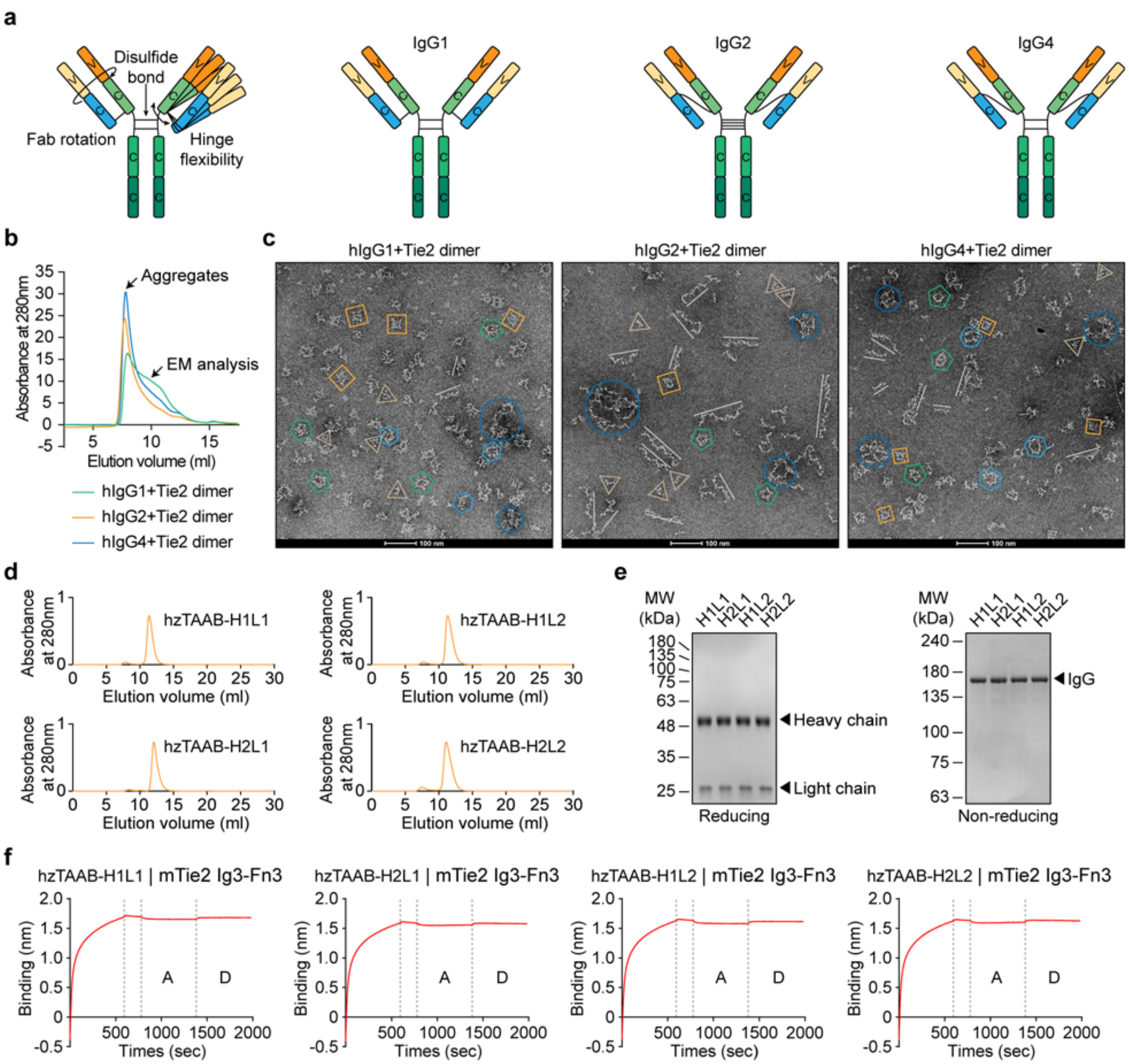

Supplementary Fig.5 | Humanized Tie2-activating antibody

a, Schematic representation of the chimeric hTAAB antibody in the form of IgG1, IgG2, and IgG4 subclasses. Interchain disulfide bonds are shown as black lines. Variable regions of heavy and light chains are colored dark orange and light orange, respectively. Constant regions of heavy and light chain are colored green and blue, respectively.

b, Size-exclusion chromatography of hTie2 Ig3-Fn3 D682C/N691C in complex with hTAAB IgGs (hIgG1, hIgG2, or hIgG4). Purified hTie2 Ig3-Fn3 dimeric mutant was incubated with hTAAB IgG1, IgG2, or IgG4 for 1 hr at a molar ratio of 2:1 (Tie2 monomer:hTAAB IgGs) and applied to size exclusion chromatography. The fraction used for negative-stain EM is indicated as EM analysis.

c, Representative micrograph of negative-stain EM analysis of Tie2 Ig3-Fn3 dimeric mutant in complex

with hTAAB IgGs (hIgG1, hIgG2, or hIgG4). Scale bars, 100 nm. The hTAAB IgGs-Tie2 dimer complex particles are classified into 6 categories (linear, <tetragonal, tetragonal, pentagonal, hexagonal, and >hexagonal form) according to their shape for quantification, and the representative particles are marked with a straight line for linear, triangle for <tetragonal, square for tetragonal, pentagon for pentagonal, hexagon for hexagonal, or circle for >hexagonal particles. Similar results were observed in *n* = 10 micrographs/group from three independent experiments.

d, Size-exclusion chromatography of IgG1-based, humanized Tie2-activating antibodies (hzTAAB H1L1, H1L2, H2L1 and H2L2).

e, Elution fractions of hzTAABs were concentrated and analyzed by SDS-PAGE and Coomassie blue staining under reducing (left) and non-reducing (right) conditions. Similar results were observed in three independent experiments.

f, Binding kinetics of hzTAABs for mTie2 Ig3-Fn3, measured by biolayer interferometry (BLI) analysis. Association (A) and dissociation (D) of mTie2 Ig3-Fn3 depicted as sensorgrams, obtained using an Octet RED96 instrument. After immobilization of the indicated antibodies on anti-human IgG Fc Capture (AHC) biosensors, association was measured by immersing the biosensors in wells containing a 1600 nM solution of mouse Tie2 Ig3-Fn3, then dissociation was measured by washing with kinetics buffer.

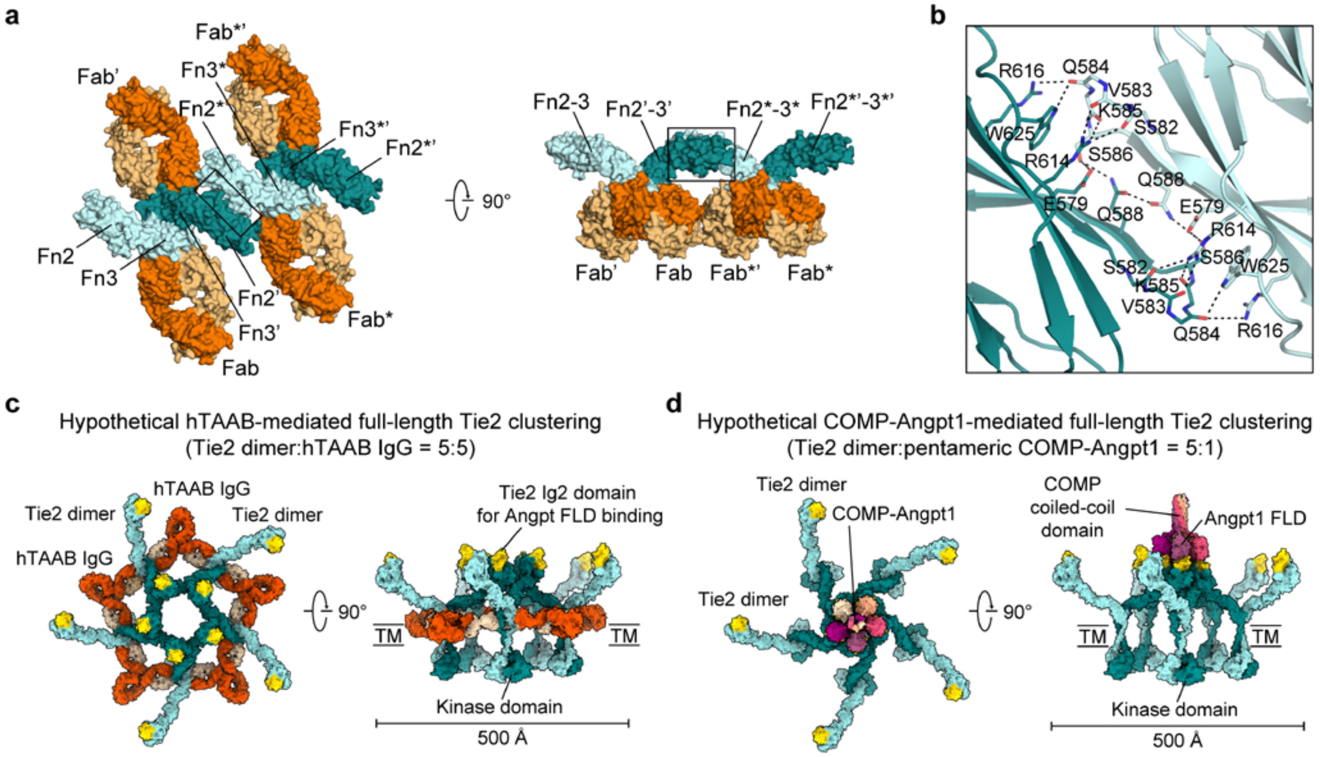

Supplementary Fig.6 | Model of full-length Tie2 clustering

a, Illustration of crystal packing interactions between two chimeric hTAAB Fab/Tie2 Fn2-3 complexes. The color schemes for chimeric hTAAB Fab and Tie2 Fn2-3 are described in **Fig. 2a**. Lateral interactions between Fn2 domains introduced by crystal packing are indicated as a black box.

b, Close-up view of crystallographic packing interactions shown in the black box in (A). The key binding residues mediated by crystal packing are shown. Hydrogen bonds and electrostatic interactions are indicated by dashed lines.

c, Proposed model for hTAAB-mediated Tie2 clustering. Cyclical higher-order structure of full-length Tie2 dimer/hTAAB IgG1 complex in a 5-to-5 assembly was modeled based on 2D class average of pentagonal closed-ring structure using the crystal structures of Tie2 Fn2-3/chimeric hTAAB Fab, Fc fragment of human IgG1 (PDB: 5VGP) and following previously reported Tie2 structures: Tie2 Ig1-Fn1 (PDB: 4K0V), Tie2 Fn1-3 (PDB: 5MYA) and Tie2 tyrosine kinase domain (PDB: 6MWE). The transmembrane domain was adopted from a previously reported NMR structure of the dimerization motif of EGFR (PDB: 5LV6).

d, Proposed model for COMP-Angpt1-mediated Tie2 clustering. Cyclical complex of full-length Tie2 dimer with COMP-Angpt1 complex in a 5-to-1 assembly was modeled based on the model of hTAAB-mediated full-length Tie2 clustering (C) using the model structure of Tie2s (C) and Angpt1 FLD/Tie2 Ig1-Fn1 complex (PDB: 4K0V) and COMP coiled-coil domain (PDB: 1VDF). (C and D) Tie2 Ig2 domains for Angpt FLD binding are colored in yellow

Supplementary Table 1 | Primer sequences for DNA cloning

Constructs	Primer	Primer sequence (5'→3')
pET-28a human Tie2 Fn2-3 (residues 541-735)	Forward	CCGCGCGGCAGCCATATGATCGGACTCCCTCCTCCAAG
	Reverse	GTGGTGGTGGTGCTCGAGTCAAGATTCTGGGAGGGTCACCAG
pBAD-chimeric_hTAAB-Fab (VL-CL/VH-CH1)	VL-Forward	GCATTTCTTCTTGCTAGCATGTTCGTTTTTTCTATTGCTACAAACGCA
	VL-Reverse	CGGTGCTGCAACGGTCCGTTTGATCTCCAGTTTTGTTC
	CL-Forward	CGGACCGTTGCAGCACCGAGCGTCT
	CL-Reverse	GCGTACGCGTTTGTAGCAATAGAAAAAACGAACATAG
	VH-Forward	TGCTACAAACGCGTACGCTCAGGTACAA
	VH-Reverse	GGGAAGACCGATGGGCCCTTCGTGGATGCGGCAGAAACTGTCACAAGGG
pOptiVEC-TOPO Chimeric_hTAAB_IgG4_HC	Forward	GATCCAACCCTTGAATTCGCCGCCACCATGGAA
	Reverse	TGGGCCCTTGGTGCTAGCGGCGCTGACAGT
pcDNA3.3-TOPO Chimeric_hTAAB_LC	Forward	GATCGAACCCTTGAATTCACTAGTGATTAATTCGCCGCC
	Reverse	TGGTGCAGCCACCGTACGCTTTATCTCCAGTTTTGT
